# Wireless Direct Microampere Current in Wound Healing: Clinical and Immunohistological Data from Two Single Case Reports

**DOI:** 10.3390/bios9030107

**Published:** 2019-09-05

**Authors:** George Lagoumintzis, Zoi Zagoriti, Mogens S. Jensen, Theodoros Argyrakos, Constantinos Koutsojannis, Konstantinos Poulas

**Affiliations:** 1 Department of Pharmacy, Laboratory of Molecular Biology & Immunology, University of Patras, 26504 Rio, Greece; zoizag@upatras.gr; 2 Institute of Physic, Danish Technical University, DK-2800 Kgs Lyngby, Denmark; Mogens.Stibius.Jensen@fysik.dtu.dk; 3 Department of Pathology, Evaggelismos General Hospital, GR 10676 Athens, Greece; lohengrin_e@yahoo.gr; 4 Department of Physiotherapy, Laboratory of Health Physics and Computational Intelligence, University of Patras, 25100 Aigion, Greece; ckoutsog@teiwest.gr

**Keywords:** chronic wounds, electrical stimulation, direct microcurrent, non-invasive, pressure ulcer, wireless technology

## Abstract

Chronic pressure ulcers are hard-to-heal wounds that decrease the patient’s quality of life. Wireless Micro Current Stimulation (WMCS) is an innovative, non-invasive, similar to electrode-based electrostimulation (ES) technology, that generates and transfers ions that are negatively-charged to the injured tissue, using accessible air gases as a transfer medium. WMCS is capable of generating similar tissue potentials, as electrode-based ES, for injured tissue. Here, through immunohistochemistry, we intended to characterize the induced tissue healing biological mechanisms that occur during WMCS therapy. Two single cases of bedridden due to serious stroke white men with chronic non-healing pressure ulcers have been treated with WMCS technology. WMCS suppresses inflammatory responses by decreasing the aggregation of granulocytes, followed by stimulating myofibroblastic activity and a new formation of collagen fibers, as depicted by immunohistochemistry. As a result, WMCS provides a special adjunct or stand-alone therapy choice for chronic and non-healing injuries, similar to electrode-based ES, but with added (i.e., contactless) benefits towards its establishment as a routine clinical wound healing regime.

## 1. Introduction

The majority of chronic (i.e., non-healing) wounds and ulcers are associated with lifestyle-related diseases such as diabetes, cardiovascular pathologies, venous stasis disease, cancer, as well as with complications caused after the treatment of life-threatening diseases, such as stroke and cerebral palsy [1,2]. Chronic wounds affect more than 10 million people worldwide, and result in enormous health care expenditures, with the total cost estimated at more than 20 billion dollars per year [3,4,5,6,7]. Chronic wounds remain a challenging problem for every clinician, with complications contributing substantially to the rates of morbidity and mortality [8]. Understanding the physiology of healing and wound care with emphasis upon new therapeutic approaches is a prerequisite for acute and long-term wound management [4].

The wound healing process consists of four major phases: (i) Hemostasis, (ii) inflammation, (iii) proliferation and (iv) tissue remodeling [9]. These phases and their biochemical outcomes must occur in the proper sequence, at a specific time, and continue for a specific duration at an optimal intensity [2].

Wounds that exhibit impaired healing, including delayed acute wounds and chronic wounds, generally have failed to progress through the normal stages of healing. Such wounds frequently enter a state of pathologic inflammation due to a postponed, incomplete, or uncoordinated healing process [10]. There are many factors that can affect wound healing which interfere with one or more phases in this process, causing improper or impaired tissue repair [2].

As a consequence, the task of proper diagnosis and the treatment of difficult-to-heal wounds for most health professionals is not a straightforward process. Indeed, health care professionals are often challenged with perplex medical conditions in the field of chronic wound healing, despite the technological innovations and the emergence of a wide range of treatments for wounds.

Besides surgical treatment, various non-surgical approaches have been developed, and numerous drugs have been introduced to help chronic wounds. These include treatments such as bandages, vacuum-assisted closure, hyperbaric oxygen and maggot debridement therapy [11]. Alternatively, radiant heat dressing, ultrasound therapy, laser treatment, hydrotherapy, electromagnetic therapy and electrotherapy are other non-surgical approaches that have a scientific basis, and have therefore been advocated in the treatment of chronic wounds [12].

In this context, there is a considerable growing body of evidence on the indication and positive effects of electrical stimulation (ES) in wound healing [13,14,15,16,17]. ES has an extensive history, with records of its use since the 17th Century [18]. ES has been shown to be effective in accelerating wound repair using different currents and types of stimulation, thus it is currently recommended as an adjunct treatment for chronic ulcers and for reinitiating or accelerating the healing process of wounds [19,20]. In fact, previous studies have shown that alternating currents (AC) are useful in managing diabetic foot ulcers [21], and direct current (DC) has shown beneficial effects in the treatment of chronic skin ulcers [19]. Furthermore, pulsed current has been reported to enhance the healing of chronic wounds [22]. High-voltage ES has shown significant therapeutic results in healing chronic ulcers by increasing blood flow and oxygen concentration around the wound, and by directing cell migration and other components of the extracellular matrix [16,17]. The usual principle for electrode-based ES implementation is to transfer the current through moist surface electrode pads that are in electrolytic contact with both the external skin surface and the wound bed. Despite the considerable evidence on the beneficial effects of electrode-based ES in wound healing, it has not been widely adopted, mostly due to pain discomfort and the increased risk of infection upon the position of the electrodes next to the wound [16].

Recently, we and others have shown the clinical beneficial effects of an innovative technology, alternative to electrode-based ES, named Wireless Micro Current Stimulation (WMCS), which belongs to the non-invasive or non-contact treatment modalities of ES [20,22]. WMCS utilizes the current-carrying capacity of charged air gas, based on the ability of nitrogen (N_2_) and/or oxygen (O_2_) molecules to accept or donate electrons, in order to distribute currents and voltages within the tissue, comparable with the electrode-based ES method (see Supplementary Materials: Calculation Model S1).

Based on that specific ability of WMCS to generate microcurrent in the tissues in a comparable manner with electrode-based ES, here we extend our previous clinical applications and findings by reporting the results of treatments of two different types of wounds, using the WMCS method. We also present important experimental evidence on the improvement of these two representative cases with chronic, non-healing, pressure ulcers of different etiology and severity. Particularly, by a series of photographs, we monitor the course of wound healing over treatment with WMCS and we record the beneficial clinical outcome of the treated cases. Furthermore, by an immunohistochemical analysis of patient’s biopsies we try to delineate the activation of the most characteristic subsequent cellular and molecular events that take place during the process of patients’ wound healing.

## 2. Materials and Methods

### 2.1. Ethical Considerations

Written informed consent was obtained from all of the legally authorized guardians of patients to participate and to share their anonymized results for evaluation and publication. Tissue samples were taken for immunohistochemical analysis. Patients could withdraw for any reason upon their guardians’ decision, without risk to their ongoing standard of care. The study was conducted in accordance with the Declaration of Helsinki, and the protocol was approved by the Ethics Committee of the University of Patras (Patras, Greece) (67257/2019).

### 2.2. Study Participants—Cases Presentation

Patients exhibited chronic, non-healing pressure ulcer wounds, persisted for ~4–6 weeks. The patients were followed clinically for adverse health events, including infection, before and after the biopsies were performed and during the Wireless Micro Current Stimulation (WMCS) treatment. Standard serial photography was used to record the wounds periodically. The detailed medical history of the two patients participating in this study is described below:

**Patient 1:** A 62-year-old white male, bedridden and unconscious for the last year after a severe stroke, presented with a great pressure ulcer at his coccyx (~11 cm × 24 cm × 8 cm deep). The ulcer arose probably due to his prolonged bed rest. Primarily, conventional wound care was adopted for the healing process (i.e., washing, disinfection, topical dressing, etc.), but the size of the ulcer gradually expanded. No history of diabetes was reported. Artificial nutrition was administered to the patient. The ulcer persisted, and it was gradually expanding for at least six weeks prior to initiation with the WMCS device. Treatment with the WMCS was initiated, using 1.5 μA current for 45 min, three times per week, for a total of ~9 months. Standard wound care by cleaning with normal saline was routine before the WMCS therapy besides its daily conventional wound care. Necrotic tissue was removed completely as well as fibrin or other coverings if necessary. During the 9-month course of treatment, systemic antibiotics were administered twice to the patient due to a common cold illness; WMCS therapy was then also paused for about a week, then continued as described above. No pain medication was administered to the patient.

**Patient 2:** A 69-year-old white male patient with a hard-to-heal bed sore ulcer on his left heel (~2 cm × 2 cm and 0.5 cm deep) participated in this study. The patient experienced a stroke three months ago, and he was gradually recovering. However, his mobility was impaired; thus, he was lying upon his bed while recovering. The ulcer appeared due to this prolonged bed rest. Conventional wound dressing was initially adopted for the healing process, but the size of the ulcer remained constant. The wound persisted—while gradually slightly expanding—for about four weeks prior initiation with the WMCS technology. Treatment with the WMCS was initiated, using 1.5 μA current for 45min daily for a total of 15 days. Standard wound care was routine before the WMCS therapy, while no topical antibacterial agent, pain medication or systemic antibiotics were administered during the course of the WMCS treatment.

### 2.3. WMCS Device and Set-Up

**Scheme 1:**
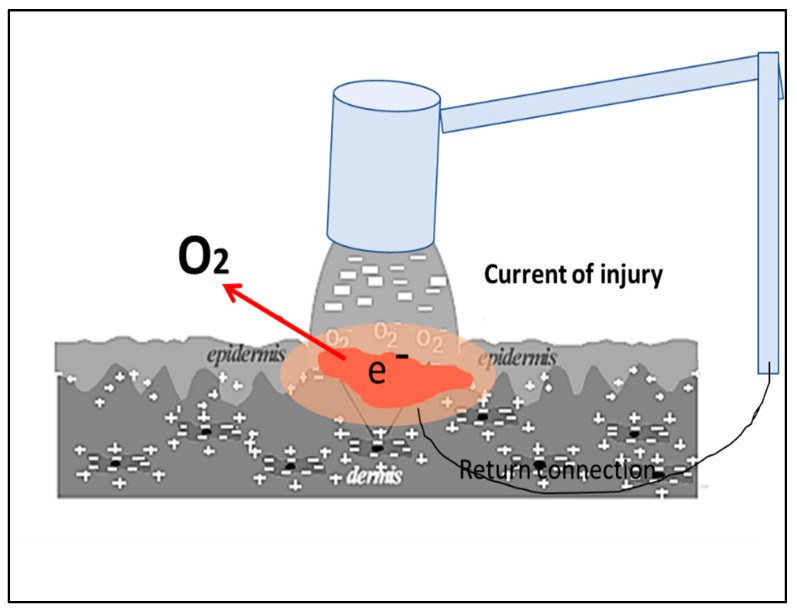
Schematic representation of the Wireless Micro Current Stimulation (WMCS) device, in a typical “spraying” of generated airborne O_2_^−^ onto a wound.

The WMCS device used in this study is a prototype based on a commercially-available model (W200, Wetling, Fredensborg, Denmark), capable of generating tissue currents and voltages as a conventional electrode-based electrostimulation (ES) device. WMCS utilizes the current-carrying capacity of charged air gas, based on the ability of O_2_ to accept or donate electrons, respectively, thus “spraying” airborne O_2_^−^ to the skin with the aid of an accelerator equipment. The device used to produce the O_2_^−^-induced current in the patient’s body is shown diagrammatically in Scheme 1, in a typical exposure condition. The flow of O_2_^−^ ions from the device is directed towards the target (i.e., the patient), who is isolated from the ground. The device is capable of producing a specific number of charged particles which are covering the area to be treated and by this way a micro-current of 1.5–4.0 μΑ intensity is generated.

The patient, while lying in bed, is connected to the WMCS device through a neutral electrode. The return path, established by wrist or ankle straps, has a dual purpose. It makes the measurement of exposure possible, and it maintains the target at virtually zero potential with respect to the ground. A control box permits the adjustment of the current and treatment duration. The intensity was set to 1.5 μΑ for all of the therapies, and the distance from the head of the device to any wound was 12–15 cm. If the head of the device was too far from the wound area for the transferred electric effect to reach the patient, there was a continuous warning tone.

### 2.4. Immunohistochemical Analysis of Biopsies

Standard—but not identical—immunohistochemical analysis was performed on each patient’s specimens. In particular, Hematoxylin and Eosin stain was done to visualize any signs of inflammation, Masson’s trichrome stain in order to clearly differentiate the collagen fibers and c-kit antigen as a marker of mast cell existence. Both Patients’ wound biopsies were taken by a qualified general practitioner at indicative time-points during the WMCS treatment. Both patients underwent a full-thickness wedge biopsy of the wound’s edge. Samples were stored in paraffin blocks, and then 3 μm histological sections were made using a histological microtome. The specimens were analyzed at the Department of Pathology, Evaggelismos Hospital, Athens, Greece, by routine semi-quantitative immunohistochemistry, as described below, and observed under a Leica DM2000 microscope. Cellular and/or protein findings were descriptively quantified without the aid of any computerized resources, by a single pathologist, in order to avoid measurement biases by multiple measurers.

### 2.5. Hematoxylin and Eosin

Slides were placed in staining jar and deparaffinized by submerging into three series of absolute xylene for four minutes, followed by 100%, 95%, 90% and 70% of ethanol for four minutes of each percentage. These slides were then washed in running tap water for two minutes.

After this, slides were submerged into Harris Hematoxylin for two minutes and then washed in running tap water for another two minutes. The slides were then submerged into 1% acid alcohol for three dips to decolorize it, and washed in running tap water for two minutes. Next, the slides were submerged into 2% potassium acetate for three minutes and again washed in running tap water for two minutes. Slides were then submerged into hematoxylin for 10 min. After that, slides were submerged into Eosin for two minutes, followed by washing in running tap water for a further two minutes. Before observation, slides were dipped into absolute xylene for one minute and finally mounted with cover slip using DPX mounting.

### 2.6. Human CD117/c-Kit 17 Antigen

Immunohistochemistry was performed on formalin-fixed and paraffin-embedded 3 μm thick sections. Tissue sections were deparaffinized in xylene and rehydrated in decreasing ethanol series. For antigen retrieval, sections were boiled in 0.01 M citrate buffer (pH = 6, slightly acidic) for 10 min. Methanol with 0.5% H_2_O_2_ was used to block endogenous peroxidase activity for 10 min. Tissue sections were then washed in Tris-buffered saline (TBS, pH = 7.6, slightly alkali) and incubated with diluted normal serum for 10 min and then treated with primary antibody for 30 min, according to the procedure outlined by the manufacturer. The applied primary antibody was c-kit monoclonal antibody (Novocastra RTU-CD117, RE 7290 k, Newcastle, UK). After rinsing with TBS, the sections were incubated with secondary antibody (Dako Envision Dual Link Labeled Polymer HRP, Glostrup, Denmark), washed again in TBS and reacted with diaminobenzine hydrochloride (DAB) for five minutes. Finally, slides were counterstained with hematoxylin and cover-slipped with a synthetic mounting media.

### 2.7. Masson’s Trichrome

For visualization of collagen fibers and the histological assessment of collagen deposition, trichrome staining was performed using the Masson Trichrome Staining Kit (Sigma Aldrich, St. Louis, MO, USA). First, the slides were deparaffinized and rehydrated through a descending manner of alcohol: 100% alcohol, 95% alcohol and 70% alcohol, for four minutes in each percentage. The slides were washed in distilled water. The slides then were submerged in warmed Bouin’s solution at 60 °C for 45 min. Next, the slides were washed in running tap water until the yellow color in samples disappeared. To differentiate nuclei, slides then were immersed in modified Wiegert’s hematoxylin for eight minutes, after that washed in running water for two minutes. In order to stain the cytoplasms and erythrocytes, the slides were submerged in anionic dyes, acid fuschin for five minutes; then again these slides were washed with running tap water for two minutes. Next, slides were treated with a phosphomolybidic acid solution for another 10 min as a mordant, and immediately the slides were submerged into methyl blue solution for five minutes in order to stain fibroblast and collagen. After that, slides were washed in running water for two minutes and lastly treated with 1% acetic acid solution for one minute. Slides then were dehydrated into a series of alcohol of 70%, 80%, 95% and 100%, respectively, for one minute within each percentage. Before observation, slides were dipped into absolute xylene for one minute and finally mounted with cover slip using DPX mounting. Finally, the slides were examined under the microscope by the same pathologist blinded to the treatments.

### 2.8. Measurement of Wound Closure

Wound closure was measured by serial standard photographs taken at several time intervals during the WMCS treatment. The target wound’s greatest length and width were measured at baseline. The ulcer size was calculated by multiplying the length and width. Percentage wound area reduction was calculated by dividing the wound area at the indicative time point after WMCS treatment with the wound area before WMCS treatment (Day = 0), and then multiplied by 100. The entire process of area measurement was done by a single person who was blinded to the group of the patient and the session for each wound being measured.

## 3. Results

### 3.1. Clinical Outcome

**Figure 1:**
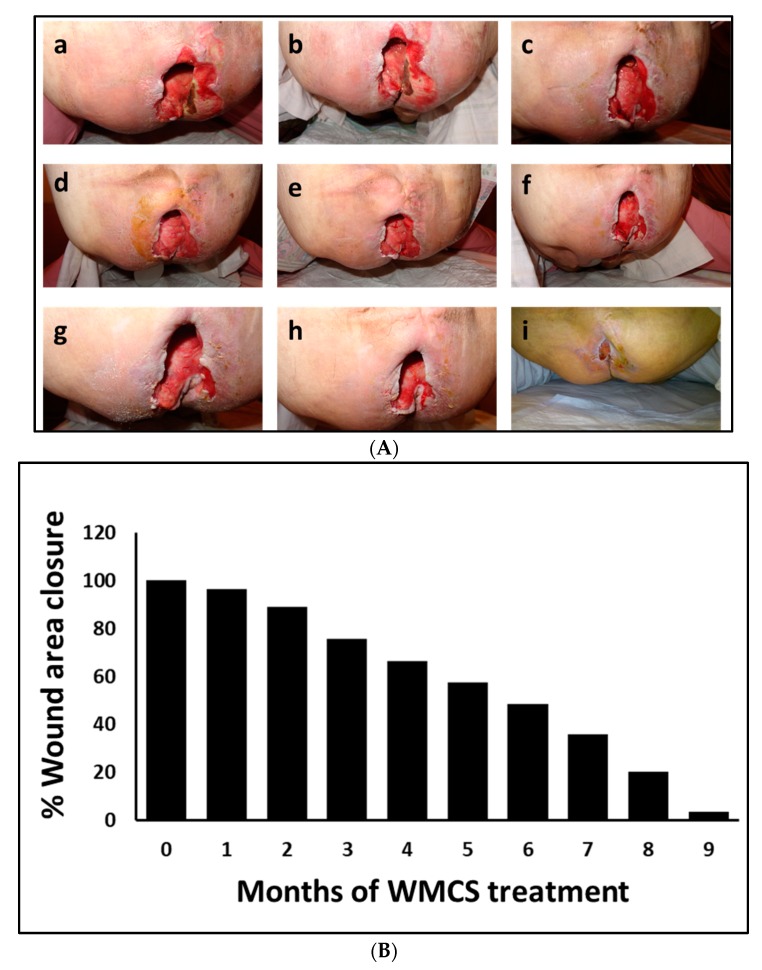
(**A**) (a–i): Serial photography of the clinical outcome of Patient 1 after WMCS treatment. (**B**) Presentation of photographic wound closure percentage of Patient 1. Wound healing is expressed as percentage wound area closure relative to original size [(wound area)/(original wound area)].

The clinical picture of Patient One’s massive pressure ulcer prior WMCS treatment is shown in Figure 1A (a–i). A clear change in wound edges with an obvious reduction in the wound’s dimensions and total area was observed after a period of ~3-month treatment Figure 1A (c). A 50% of wound closure was achieved after a 6-month period of treatment Figure 1A (d–f), while a more than 90% wound closure and regrowth of normal skin was seen by the end of a total of 8 to 9 month WMCS treatment Figure 1A (g–i).

Measurement of the wounded area is one of the key aspects in the assessment of the healing process, since it can indicate healing improvement. Figure 1B shows the percentage (%) of wound closure for Patient 1 from photographs taken immediately after treatment with WMCS. As shown, wound healing started gradually after WMCS treatment, with an obvious macroscopic improvement even at the end of the 1st month of treatment. At approximately six months of treatment, a 50% wound closure was achieved, while by the end of the 9-month treatment a greater than 95% of wound closure was observed as compared to the initial wound area before WMCS treatment.

**Figure 2:**
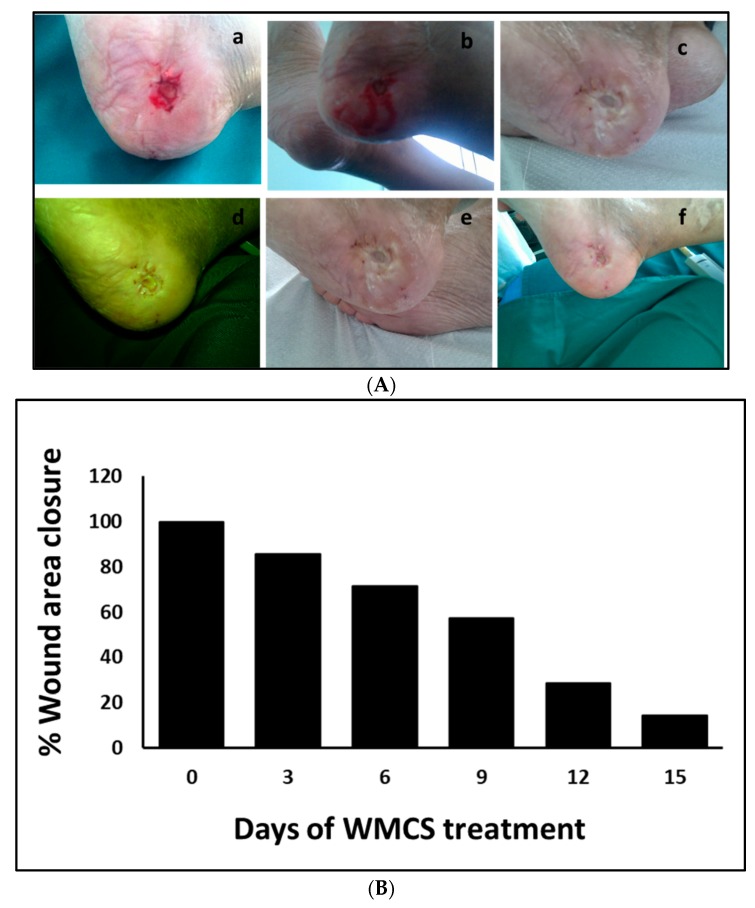
(**A**) (a–f): Serial photography of the clinical outcome of Patient 2 after WMCS treatment. (**B**) Presentation of the photographic wound closure percentage of Patient 2. Wound healing is expressed as percentage wound area closure relative to original size [(wound area)/(original wound area)].

For Patient 2, the clinical picture of the wound prior WMCS treatment is shown in Figure 2A (a). After three sessions, healthy new epithelial tissue was visible in the wound bed and at the wound edge, Figure 2A (b). A clear reduction in the wound’s dimensions and total area was achieved after 6 sessions, Figure 2A (c), allowing for no dressing changes. Continued decrease in scar tissue and regrowth of normal skin were observed from day 9 to day 15, Figure 2A (d–f), with improved wound contraction and significant re-epithelization.

Figure 2B is showing the percentage (%) of wound closure for Patient 2 as a factor of time treatment with the WMCS technology from photographs taken after treatment. As we can see, this wound started gradually to heal immediately after WMCS treatment, with more obvious changes in wound dimensions after a week (Day 6 to 7). An approximately 50% of wound area reduction in size was observed at day 8 to 9, whereas at day 15 with WMCS treatment, the wound was almost entirely healed.

### 3.2. Immunohistochemical Analysis

**Figure 3:**
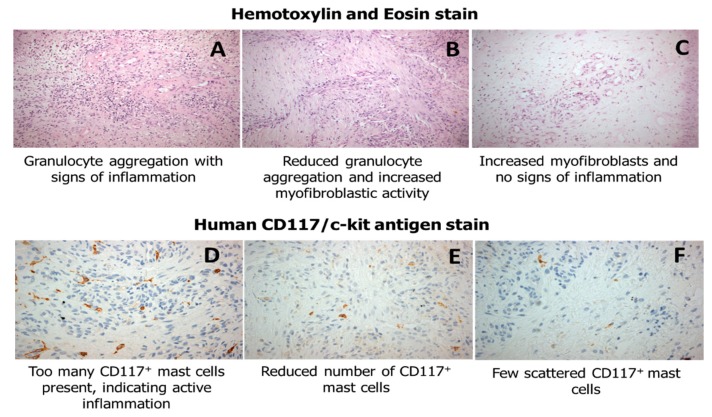
(**A**–**C**) Hemotoxylin and Eosin immunohistochemistry of tissue biopsies from Patient 1, demonstrating initial granulation tissue formation and inflammation markers and increased myofibroblastic activity, and (**D**–**F**) CD117+ mast cells after WMCS treatment.

Monitoring healing progress by tissue biopsies of the wounded area is the hallmark of the evaluation of an active and proper wound healing process. Thus, tissue biopsies were immunohistochemically analyzed in order to identify potential cellular events upon WMCS treatment that are indicative of a restart of wound healing. Figure 3A–C shows the Hematoxylin and Eosin stain of tissue biopsy from Patient 1. In detail, Figure 3A depicts signs of active granulocyte aggregation with some signs of inflammation after 1-month WMCS treatment sessions. A suppression of inflammation upon WMCS treatment (2-month) as evidenced followed by an increase in myofibroblastic activity and a reduced granulocyte aggregation, is demonstrated in Figure 3B. After 3-month of WMCS treatment (Figure 3C), microscopic examination revealed further established myofibroblastic activity and almost totally diminished granulocyte aggregation.

Increased numbers of mast cells (c-kit antigen stain, panels D–F) are present at early stages of wound healing, indicating an active process of inflammatory reactions (Figure 3D). WMCS treatment seems to affect the activation of mast cells, as an increased number of visible mast cells is observed after 1-month WMCS treatment (Figure 3D), ultimately leading to the observation of few scattered mast cells between 2-month and 3-month of WMCS therapeutic treatments (Figure 3E,F).

**Figure 4:**
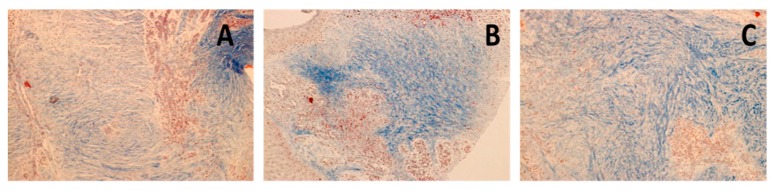
(**A**–**C**) Masson’s trichrome stain of tissue biopsies from Patient 2. Masson’s stain indicating collagen formation and myofibroblastic proliferation after WMCS treatment.

Tissue biopsies from Patient 2 were stained apart from H&E stain with the Masson’s trichrome stain and the c-kit antigen for collagen formation, myofibroblastic proliferation and mast cell existence after WMCS treatment. As shown in Figure 4A–C, a gradually increased amount of collagen deposition during the healing process is observed after WMCS treatment.

**Figure 5:**
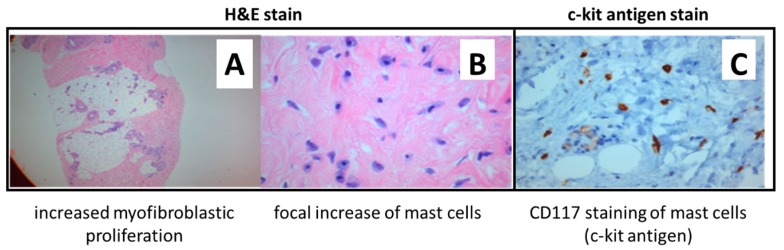
(**A**–**C**) Immunohistochemical analysis of tissue biopsies from Patient 2, showing increased myofibroblastic activity, with a relative focal increase of mast cells (H&E stain 5A and 5B, respectively), and c-kit (CD117) antigen stain showing reduced CD117+ mast cells over WMCS treatment (5C).

Moreover, WMCS enhances myofibroblastic proliferation (Figure 5A), yet with a relative focal increase of mast cells after three Days (Figure 5B). Finally, after six Days of WMCS treatment few scattered mast cells are seen as revealed by the c-kit antigen (Figure 5C).

## 4. Discussion

Pressure ulcers, also called bedsores or decubitus ulcers, develop when there is too much unrelieved pressure on the skin in combination with shear and/or friction. They form typically over a bony prominence, leading to ischemia, cell death and tissue necrosis [23,24]. They are more common in bedridden patients, affecting their quality of life, morbidity and mortality. Once a pressure ulcer develops, not only does it cause pain and discomfort, but it may lead to complications such as infection, like meningitis, cellulitis and endocarditis, with the potential for sepsis and death [25]. The shoulder blades, tailbone, elbows, heels and hips are the most common sites for bed sores because these areas contain little muscle and fat [26]. The development of a pressure ulcer can interfere with functional recovery, promote social isolation, and contribute to excessive length of hospital stay [27]. As a consequence, they have been described as one of the costliest and physically devastating complications [28,29], and they are the third most expensive disorder after cancer and cardiovascular diseases [30].

Treatment of pressure ulcers involves multiple methods intended to alleviate the conditions contributing to ulcer development (support surfaces, repositioning and nutritional support), protection of the wound from contamination, and the creation of a clean wound environment, promotion of tissue healing (local wound applications, debridement and wound cleansing), adjunctive therapies, as well as consideration for surgical treatment [31]. Understanding the function of bioelectricity in wound healing offers a possibility for the therapeutic implementation of ES, especially when natural regeneration mechanisms have been paused. ES is a well-proven technique for the therapy of chronic wounds [32]. Its application for the treatment of wounds adopts the physiological fact that skin and epithelia act as batteries and have electric potentials.

When a wound occurs in the epithelium, there is an electrical leak that short-circuits the skin, enabling the current to flow out of the wound, resulting in the current of injury. This leak of current provides signals for the migration of the epithelia into the wound, initiating a physiological endogenous regeneration processes [32,33,34]. Disruption of these physiological electric fields and ionic currents alters normal organ development, tissue regeneration, and thus, proper wound healing [35,36,37].

Our team has previously experimented on WMCS, an innovative modality of traditional electrode-based ES, and we have particularly shown recently that WMCS can be an effective method of diabetic-related wound care [22], for difficult to treat chronic wounds [20], for firework burns [38], as well as for Martorell’s ulcer [39]. Tissue potentials and currents generated by either electrode-based ES or WMCS seem to be the determinant factor which initially drives the beneficial clinical outcomes of these two similar, but distinct, techniques.

In this report, based upon clinical as well as on immunohistochemical evidences, we further report on WMCS treatment with two single case reports of distinct pathologies. Our results showed that WMCS aids the wound healing of pressure ulcers by restoring the natural current of injury, thus, reinitiating the body’s tissue physiological regeneration mechanisms. Notably, WMCS suppresses inflammatory reactions by reducing granulocyte aggregation, followed by stimulating myofibroblastic activity and collagen fiber formation. These results are in accordance, not only with our previous clinical observational studies, but also to others, who have effectively used similar ES modalities to treat pressure ulcers [40,41,42]. In addition, several recent systematic reviews provide positive recommendation regarding the effectiveness of ES to increase wound healing [43,44,45,46,47,48]. Consequently, based on literature as well as on our previous work, WMCS offers a unique treatment option to chronic and non-healing wounds of diverse etiopathology. WMCS in particular, is believed to aid in wound healing by imitating and restoring the natural electrical current that is been disrupted in injured skin.

## 5. Conclusions

WMCS has been both theoretically, clinically and experimentally, evidently proven as an effective, easy-to-use, non-invasive and time-efficient treatment for the wound healing of different etiopathologies, as electrode-based ES; however, with more of its advantages reflecting its contactless application. Yet, greater knowledge of the specific repairing mechanisms upon WMCS treatment will convey us toward more effective and broad clinical applications. To this end, more experimental and clinical efforts are needed to elucidate how wound regeneration is influenced by the choice of optimal parameters of WMCS (i.e., polarity, frequency, duration) that could affect the general healing process in each medical case.

## Supplementary Materials

The following are available online at https://www.mdpi.com/2079-6374/9/3/107/s1, Calculations S1: Calculation Model of Tissue Potentials Between WMCS and (traditional) Electrode-Based ES Method.
