# Supplementary material for: Wireless Direct Microampere Current in Wound Healing: Clinical and Immunohistological Data from Two Single Case Reports
Source: Biosensors (Basel). 2019 Sep 5;9(3):107. doi: 10.3390/bios9030107 (PMC6784371; doi:10.3390/bios9030107)
Supplement: Supplementary file 1 [file biosensors-09-00107-s001.pdf]

Supplementary Materials

Wireless Direct Microampere Current in Wound Healing: Clinical and Immunohistological Data from Two Single Case Reports

George Lagoumintzis [1,*], Zoi Zagoriti [1], Mogens S. Jensen [2], Theodoros Argyrakos [3], Constantinos Koutsojannis [4] and Konstantinos Poulas [1,*]

1 Department of Pharmacy, Laboratory of Molecular Biology & Immunology, University of Patras, 26504 Rio, Greece; zoizag@upatras.gr
2 Institute of Physic, Danish Technical University, DK-2800 Kgs Lyngby, Denmark; Mogens.Stibius.Jensen@fysik.dtu.dk
3 Department of Pathology, Evaggelismos General Hospital, GR 10676 Athens, Greece; lohengrin_e@yahoo.gr
4 Department of Physiotherapy, Laboratory of Health Physics and Computational Intelligence, University of Patras, 25100 Aigion, Greece; ckoutsog@teiwest.gr
* Correspondence: glagoum@upatras.gr (G.L.); kpoulas@upatras.gr (K.P.); Tel.: +30-2610-969953 (K.P.)

Received: 17 July 2019; Accepted: 2 September 2019; Published: 5 September 2019

Calculation Model S1: Calculation Model of Tissue Potentials Between WMCS and Electrode-Based ES Method.

Herein, using an appropriate physicomathematical model, Prof. M. Jensen proves that the WMCS method compared with the electrode-based ES, yields to qualitative similar results in their physiological effects in tissues.

Calculation of Tissue Electrical Potentials and Voltages by ES and WMCS

The electrical potential (φ) and the current density (j) inside the tissue depend on the shape and position of the electrodes, the conductivity (σ) of the tissue, and the geometry of the body. In each area of tissue where σ is constant, Laplace's equation (Δφ = 0) must be met with the appropriate boundary conditions. This can typically be calculated by numerical methods, *e.g.* calculations of finite elements. However, analytical solutions can be provided for simple geometries and constant conductivity throughout the entire tissue region. Since we want to compare when two small plate electrodes supply the current with the WMCS method, where the current is "sprayed" at an almost constant current density to a given tissue region, we believe that by studying these analytical solutions we can gain more insight into the subject than with numerical numbers.

We will therefore consider a case in which the semi- infinite area of z > 0, bounded by the plane z = 0, is filled by a tissue of constant conductivity, σ. For z → ∞, the potential is taken to be 0. The current is supplied through a small disk of radius a, around (0, 0, 0), with the other electrode far away, *i.e.*, at infinity, where φ = 0.

In case A, the disk is given a fixed voltage, V0, whereas in case B, a current with constant current density, J_0, is supplied over the disk. Case A models the usual electrode-based ES method and case B the WMCS method. Both methods have cylindrical symmetry around the z-axis, and the solution for φ can therefore be given in cylindrical coordinates (r, θ, z), where r is the distance to the z-axis, θ is the polar angle, and z is the distance from the surface. In both cases A and B, the solution for z ≥ 0 has the form:

$$\varphi = \int_0^{\propto} f(s) J_0(sr) e^{-sz} ds \quad (1)$$

Where J_0 is a Bessel function of order zero, and f(s) is a function that must be chosen such that φ satisfies the boundary conditions for z = 0.

In case A, the boundary condition for z = 0 are: φ = V0 for 0 ≤ r ≤ a, and the current density, jz = $-\left(\sigma \frac{\vartheta\varphi}{\vartheta z}\right)$ z=0 = 0, through the plane z = 0 or r > a. This will be fulfilled when f(s) is given by:

$$fA(s) = \frac{2Vo}{\pi s} \sin(s\alpha) \quad (2)$$

For this value of f(s), the integral (**1**) can be expressed by the elementary function:

$$\varphi A = \frac{2V_0}{\pi} \text{Arc sin}\left(\frac{2\alpha}{\sqrt{(r-a)^2+z^2+\sqrt{(r+a)^2+z^2}}}\right) \quad (3)$$

The total current, IA, in case A can now be found by integrating the current density, jz, over the disk-shaped electrode with Voltage V0:

$$IA = 2\sigma V0 \int_0^{\alpha}\left(\int_0^{\infty} \sin(\sigma\alpha) J_0(sr)ds\right) r2\, dr = 4\sigma\alpha V0 \quad (4)$$

In case B, f(s) must be: $fB(s) = \frac{j_0\alpha}{\sigma}\frac{J_1(s\alpha)}{s}$, ($J_1$ is the Bessel function of order 1) which gives:

$$\varphi B = \frac{J_0\alpha}{\sigma}\int_0^{\infty} e^{-sz}\, J0\,(sr) J1(s\alpha)\frac{ds}{s} \quad (5)$$

The integral in equation (**5**) cannot be given in an elementary form when z > 0, but can be expressed as an infinite sum of hypergeometric functions.

For z = 0, φB is given by single hypergeometric functions. The average value of φB over the disk r < a, for z = 0, is found to be $V\alpha v = \frac{8J_0\alpha}{3\pi\sigma}$, and the maximal value $\varphi B\ max = \frac{J_0\alpha}{\sigma}$.

Because the total current is IB = πa2j0, the ratio of the current to the average voltage is: IB/Vav = $\frac{3\pi^2\ \sigma a}{8}$, whereas in case Awe get: IA/V0 = 4σα.

Measurement of Electrical Potentials and Currents

Graphs 1 and 2 demonstrate the electrical potential values at different r and z values in theoretical cases A and B. When we compare the figures for theoretical cases A and B, no significant differences can be seen. It should be noted, however, that an electrode is usually much smaller than the wound, whereas the current at the WMCS method is supplied over a larger area. This means that a ≈ 1 mm in case A, whereas it can easily be 10 mm or more in case B. From the expressions for the total currents, we see that for the same current in cases A and B, the ratio between V0 and φB max is V0/φB max = πaA/(4aB) ≈ 10.

Graphs

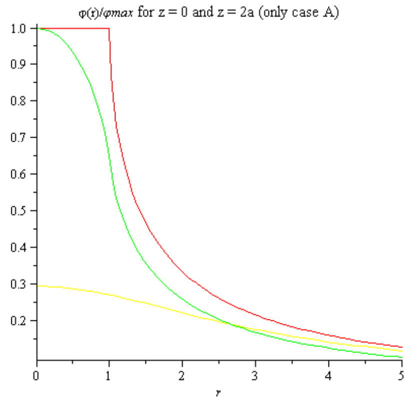

Graph 1: φ/φ_{max} as a function of r/a for z = 0 (case A, red; case B, green) and z = 2a (case A, yellow).

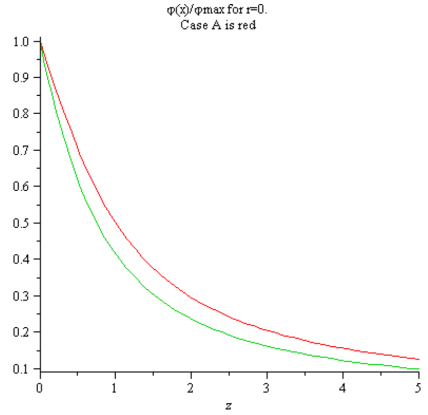

Graph 2: φ/φ_{max} as a function of z/a for r = 0 (case A, red; case B, green).
